# Depot-specific differences in visceral and subcutaneous adipose tissue from patients with obesity

**DOI:** 10.1080/21623945.2026.2708374

**Published:** 2026-07-26

**Authors:** Mathis Neuhaus, Claes Fryklund, Min Cai, Johan Fernø, Gunnar Mellgren, Carolina E. Hagberg, Karin G. Stenkula

**Affiliations:** a Department of Experimental Medical Science, Lund University, Lund, Sweden; b Division of Cardiovascular Medicine, Department of Medicine Solna, Karolinska Institutet and the Center for Molecular Medicine, Karolinska University Hospital, Stockholm, Sweden; c Hormone Laboratory, Department of Medical Biochemistry and Pharmacology, Haukeland University Hospital, Bergen, Norway; d Mohn Research Center for Diabetes Precision Medicine, Department of Clinical Science, University of Bergen, Bergen, Norway

**Keywords:** Adipose tissue biology, extracellular matrix, collagen, cell size, adipose depot differences, lipolysis, glucose transport

## Abstract

Factors limiting fat cell expandability remain scarcely explored, and comprehensive functional comparisons of adipose depots in humans are limited. In the present study, we collected visceral adipose tissue (VAT) and subcutaneous adipose tissue (SAT) paired-samples from 22 patients with obesity undergoing elective Roux-en-Y gastric bypass surgery. Whole tissue samples were used to determine adipocyte cell size using a coulter counter and gene expression levels using bulk mRNA sequencing. Isolated adipocytes from the same tissue samples were used to functionally assess glucose uptake using glucose tracer assay, and lipolysis by measuring glycerol release. Western blotting was used to determine Caveolin 1 levels in adipocytes. The relationship between *COL3A1* expression and systemic health outcomes was assessed using data from the Adipose Tissue Knowledge Portal. We found that SAT adipocytes were larger in size, displayed higher basal lipolysis and higher lipolysis-related gene expression than VAT adipocytes. Isoprenaline-induced lipolytic responsiveness, as well as basal and insulin-stimulated glucose uptake were higher in VAT than in SAT adipocytes. SAT displayed higher levels of extracellular matrix components associated with cellular flexibility and higher cellular Caveolin 1 levels than VAT. Further, we identified subcutaneous adipose tissue *COL3A1* as a potential contributor to insulin resistance in obesity, positively associated with BMI, HOMA-IR and adipocyte size. In summary, our findings underscore distinct metabolic properties of subcutaneous and visceral adipocytes in obesity, with SAT adipose tissue exhibiting characteristics that possibly promote greater cellular expandability.

## 1. Introduction

Obesity is characterized by an erratic expansion of white adipose tissue (WAT) and is a well-established risk factor for insulin resistance and type 2 diabetes [1]. Subcutaneous white adipose tissue (SAT) and visceral white adipose tissue (VAT) comprise the two largest adipose tissue storage sites in the human body [1]. SAT and VAT exhibit crucially different structural and metabolic characteristics that also differ from their counterparts in mice [2,3]. Importantly, despite SAT constituting most of the human fat mass, changes in VAT morphology and function have repeatedly been shown to be more strongly correlated with obesity-related co-morbidities [4,5].

Adipose tissue expansion in the course of obesity is associated with a drastic increase in adipocyte size in both depots [6]. Hypertrophic adipocytes are in general more insulin resistant and display elevated spontaneous lipid release but decreased hormone-stimulated lipolysis [7,8]. Regional differences in fat cell size are determined by factors like species, grade of obesity, biological sex, age, and ethnicity [8]. In general, human VAT displays smaller adipocyte size than SAT [8]. VAT is generally portrayed as more metabolically active, reflected by higher lipolytic activity, reduced antilipolytic responsiveness to insulin, and direct portal drainage to the liver of VAT compared to SAT [9–12]. Yet, the higher absolute SAT mass likely leads to a superior contribution to circulating free fatty acids levels [13]. VAT is more prone to immune cell infiltration and fibrosis during weight gain and displays a more pro-inflammatory adipokine secretion pattern [14–16]. In support of depot-specific differences, studies exploring expression of extracellular matrix (ECM) components and fibrotic changes in the obese state have yielded distinct results comparing SAT and VAT [17–19]. SAT, primarily lower body SAT, functions as a long-term lipid sink with greater adipogenic capacity in its progenitor cells, leading to more effective triglyceride accumulation [20,21]. Notably, independent of depot origin, erratic adipocyte size expansion is generally associated with impaired cellular insulin response and dysfunctional lipid metabolism [8]. While some of these factors clearly contribute to the fact that hypertrophy of human visceral adipocytes is more strongly associated with metabolic dysfunction and insulin resistance than that of subcutaneous adipocytes, several discrepancies remain. Factors enabling and restricting this expansion and lipid-storage capacity, and how those relate to insulin resistance, remain largely unknown.

Herein, we set out to characterize subcutaneous adipocytes and visceral adipocytes derived from patients with obesity undergoing elective Roux-en-Y gastric bypass surgery, with a focus on paired fat-depot comparison of adipose tissue extracellular matrix composition and cellular characteristics related to adipocyte expandability and its relationship with insulin resistance.

## 2. Materials and methods

### 2.1. Patient characteristics and ethical statement

Bariatric Surgery on the West Coast of Norway study enrolled individuals aged 18–60 years eligible for bariatric surgery based on national recommendations (BMI≥40kg/m^2^ or BMI≥35kg/m^2^ with at least one weight-related condition as hypertension, diabetes mellitus type 2 and/or sleep apnoea). The exclusion criterion was pregnancy during the study period. Blood samples were collected after an overnight fast, and serum analyses were performed according to the hospital’s standard procedures. Adipose tissue samples were obtained during surgery from the omental adipose tissue depot and the abdominal-subcutaneous depot. The study was approved by the Regional Committee for Medical Research Ethics, Western Norway (REK 2010/502 and 2015/2343), conducted in accordance with the revised Declaration of Helsinki, and written informed consent was obtained from all participants before inclusion Table 1.

**Table 1.** Patient characteristics.

	Number	Mean	± SD
Patient number	22		
Bypass|Sleeve	2|20		
Smoking|Non-smoking	2|20		
Men|Women	4|18		
Age (years)		41.82	11.08
BMI (kg/m^2^)		39.62	2.90
Weight (kg)		117.65	16.72
Height (m)		1.72	0.10
Hba1c (mmol/mol)		34.86	4.10
HOMA-IR		3.57	2.01
Total Cholesterol (mM)		4.46	0.83
LDL (mM)		3.01	0.68
HDL (mM)		1.13	0.25
Triglycerides		1.39	0.54
CRP (mg/L)		5.32	4.32
Glucose (mM)		5.66	0.77
Insulin (µIU/ml)		13.78	6.81

Homoeostasis Model Assessment of insulin resistance (HOMA-IR) was calculated as (fasting plasma glucose in mmol/L x fasting serum insulin in mIU/L)/22.5 [22]. Body mass index (BMI) was calculated as weight (kg) divided by height squared (m^2^).

### 2.2. Adipocyte isolation

Primary adipocytes were isolated from visceral and subcutaneous adipose tissue depots in Krebs Ringer Bicarbonate HEPES (KRBH) buffer, pH 7.4, containing 200 nM adenosine, 3% (w/v) bovine serum albumin (BSA, 81–053-1 Millipore), and 1 mg/ml collagenase type I (Worthington Biochemical Corporation collagenase type I, LS004194) using an established protocol [23]. Collagenase digestion was performed at 37°C and 120 rpm in a shaking incubator for approximately 30 minutes. Isolated cells were filtered through a 400 µm nylon-mesh and washed three times in KRBH buffer containing 200 nM adenosine and 3% (w/v) BSA. Floatation times between washes were limited to 5–7 minutes. Cells were then allowed to recover ~15 min prior further analysis. Adenosine was included in the buffer to suppress spontaneous lipolytic activity and cell breakage of adipocytes.

### 2.3. Cell size distribution

Subcutaneous and visceral adipose tissue samples were cut into 4–5 pieces of around 5 mg. Adipose tissue pieces were subsequently fixed for about one week in 2%-osmium tetroxide diluted in a buffer containing 0.2 M 2,4,6-Trimethyl Pyridine (Sigma C0505), 25 mM HCl and 0.9% NaCl. Osmium-fixed cells are then subsequently washed trough a 25 µm and 250 µm meshes and collected in 0.9% NaCl solution. Particle sizes of osmium fixed particles were determined using a Multisizer 4e Coulter Counter (Beckman-Coulter, Brea) with a 400 µm aperture. The range of cell sizes that can effectively be measured using this aperture is 20–240 µm. The instrument was set to count 6000 particles, and the suspension of osmium-fixed particles in 0.9% NaCl was diluted in Beckman Coulter Isoton II Diluent (Beckman Coulter C96980), so that coincident counting was <10%. For each sample two independent runs, each counting 6000 particles, were performed and averaged. Conclusively, this approach allowed us to collect information about the sizes of particles for each adipose tissue sample. The pulse size per particle were expressed using particle diameters and displayed as histograms of counts against diameter using linear bins and a linear scale for the x-axis. Small adipocytes were defined as the cumulative frequency % of cells smaller than the diameter of the nadir [24]. The nadir is defined as the local minimum of the frequency percentage in between the bimodal cell-size distribution [24,25]. The nadir acts as a cut-off point separating the smaller adipocyte population from the larger adipocyte population. Large adipocytes are herein defined as fat cells whose diameter is above the nadir. Peak of large cells was defined as the cell diameter of the peak (highest frequency %) in cells larger than the nadir [24,25]. In other words, the most common diameter (mode) among the larger adipocytes. For more information about these metrics see also [25]. The average volume of an adipocyte was calculated from the mean cell diameter measured by the Coulter counter, assuming that the cells were spherical. This estimated average cell volume was then used to calculate how many adipocytes were present in a fixed volume of cell suspension. Glucose uptake and lipolysis measurements were subsequently normalized to this estimated cell number, allowing the results to be expressed per cell rather than per total suspension volume.

### 2.4. Glucose uptake

Glucose uptake was determined as previously described [26]. Briefly, collagenase-isolated adipocytes (10% v/v) were incubated in KRBH buffer supplemented with 200 nM adenosine and 3% (w/v) BSA with or without insulin (10 nM), for 30 min in technical triplicate, followed by the addition of 14C-D-glucose (0.5μL/mL, NEC042) which is taken up and metabolized by adipocytes, and an additional 30-min incubation in total volume of 500 µl. The adipocytes are separated from incubation media by centrifugation (~10 000xg, ~10s) of 300 μL of each cell suspension in microtubes containing 80 μL dinonylphtalate oil. The adipocyte fraction was collected from the top layer, dissolved in scintillation fluid (Optima Gold, Perkin Elmer), and subjected to scintillation counting. The amount of glucose taken up by the adipocytes is assessed by measuring radioactive decay. Cytochalasin B, which blocks all glucose transport was used to estimate non-specific radiolabeled glucose binding to the cell surface. Glucose uptake measurements were normalized to the estimated number of cells based on coulter counter analysis and insulin responsiveness was calculated as the fold response of insulin over basal glucose uptake. Insulin induced a significant increase in glucose uptake in both VAT (*p* < 0.0001) and SAT (*p* < 0.0001) adipocytes.

### 2.5. Lipolysis

Cellular lipolysis was determined by measuring the release of glycerol by adipocytes into the incubation media. Briefly, collagenase-isolated VAT and SAT adipocytes [10% (v/v) cell suspension] were treated with or without isoprenaline (ISO; 10 nM Sigma I5627) for 30 min in KRBH buffer containing 200 nM adenosine and 3% (w/v) BSA in a total volume of 400 µl. After incubation, medium samples were removed for enzymatic determination of the glycerol content. In short, 100 μl free glycerol reagent (F6428, Sigma) was added to 30 μl of medium sample. Absorbance at 540 nm was measured in a FLUOstar Omega microplate reader (BMG Labtech, Ortenberg, Germany) after incubating for ~15 min at 37°C. Each sample was analysed in technical duplicates. Glycerol release measurements were then normalized to the estimated number of cells in the assay. Isoprenaline responsiveness was calculated as the fold response of isoprenaline over spontaneous glycerol release. Isoprenaline induced a significant increase in glycerol release in both VAT (*p* < 0.0001) and SAT (*p* = 0.0099) adipocytes.

### 2.6. Western blot analysis

Collagenase-isolated adipocytes were washed three times in BSA-free KRBH, and floating cells were lysed 1:1 in a lysis buffer containing 50 mM Tris/HCl pH 7.5, 1 mM EGTA, 1 mM EDTA, 0.27 M sucrose, 1% NP-40, and a complete protease and phosphatase inhibitor cocktail (Roche, Basel, Switzerland). Lysates were centrifuged for 10 min at 13,000 g, protein lysates were collected from the infranatant, and bradford measurement was used to determine the total protein content. Equal amounts of protein lysates (10 µg according to Bradford measurement were loaded on stain-free TGX 4–15% gradient gels (No. 5,678,084, Bio-Rad, Hercules) in a sample buffer containing 1X Laemmli Sample Buffer (BioRad, #1610747) and 300 mM 2-Mercaptoethanol (BioRad, #1610710). Separation of proteins was performed at 200 V for 1 h in Tris/Glycine/SDS Electrophoresis Buffer (BioRad, #1610772EDU). After separation, the stain-free gel was activated for 45s in a Bio-Rad ChemiDoc MP Imaging System, and proteins were transferred to an Immun-Blot® Low Fluorescence PVDF membrane (BioRad, #1620260). The stain-free signal on the membrane was measured immediately after transfer and subsequently used for total protein normalization. The membrane was then blocked in 5% skim-milk powder for 1 h at room temperature, washed in TBST-0.1% Tween20, and incubated in primary antibodies at 4°C overnight. The primary antibody against caveolin 1 (Cell Signalling Technology Cat# 3267, RRID:AB_2275453) was used in a 1:400 000 dilution. Secondary antibody incubation was performed for 1 h at room temperature with anti-rabbit (No. 314,060, Thermo Fisher Scientific, Waltham) horseradish peroxidase-conjugated secondary antibody diluted 1:2500. After primary and secondary antibody incubation, membranes were washed for 5 × 5 min in TBST-0.1% Tween20. Enhanced chemiluminescence reagent (SuperSignal West Pico Chemiluminescent Substrates, Thermo Scientific) was used for developing the signals. Bio-Rad ChemiDoc MP Imaging System and Image Lab software (Bio-Rad Laboratories) were used for detection and quantification.

### 2.7. RNA sequencing

Eighteen paired, whole-tissue samples of visceral adipose tissue (VAT) and subcutaneous adipose tissue (SAT) were homogenized in QIAzol lysis reagent (Qiagen, 79,306) using a QIAGEN TissueLyser at 30 Hz for 3 min. Chloroform was then added to enable phase separation into aqueous and organic fractions. Total RNA was extracted using the RNeasy Mini Kit (Qiagen, 74,104) according to the manufacturer’s instructions. Bulk mRNA sequencing was then performed by Novogene (Novogene (UK) Company Limited). Sample purity, integrity and quantity were assessed prior to sequencing using an Agilent 5400 and samples included for sequencing had RNA integrity values of RIN ≥ 6.0. Sequencing, quality controls and preliminary data pre-processing were performed by Novogene. Briefly, sequencing was performed on a NovaSeq platform using 150-bp paired-end reads, with a target output of 15 GB per sample. Raw sequencing reads in FASTQ format were processed using fastp to remove adapter-contaminated reads, reads containing poly-N sequences, and low-quality reads. Sequence alignment was conducted with HISAT2 (2.2.1) against the human reference genome GRCh38/hg38. To assess the number of reads that align to genomic features, HTSeq-count was used. For differential expression analysis, raw gene-level counts were analysed in R v4.3.3 using DESeq2 (1.42.0). Because VAT and SAT samples were paired within the same participants, participant ID was included in the design formula to account for within-subject pairing. DESeq2 default normalization and dispersion estimation procedures were used. DESeq2’s default independent filtering based on mean normalized counts was applied. Potential batch effects and relevant covariates were considered during exploratory analysis, specifically using PCA plots and heatmaps. Based on the exploratory analysis, we concluded that the effects of these factors were neglectable and only subject and depot were included in the final model. Genes were considered differentially expressed using an Benjamini – Hochberg adjusted *p*-value threshold of FDR < 0.05 and an absolute log2 fold-change threshold of >1. PCA was performed using the 5,000 most variable genes from the variance-stabilized count matrix to assess sample clustering. KEGG pathway enrichment analysis was performed using the clusterProfiler package. Differentially expressed genes were defined by a Benjamini – Hochberg-adjusted *p* value < 0.05 and an absolute log2 fold change ≥ 1. Additional information on sequencing depth, mapping rates, and other quality-control metrics is available in the supplementary files (http://doi.org/10.6084/m9.figshare.32090950).

### 2.8. Statistical analysis

Statistical analyses were performed using R version 4.5.2 [27] and GraphPad Prism 10 (GraphPad Software) software as indicated in figure legends. Assumptions of linearity, homoscedasticity, and gaussian distribution were assessed prior analysis. Simple linear regression (GraphPad Prism), repeated-measures two-way ANOVA and post-hoc multiple comparisons (R using the ‘afex’ and ‘emmeans’ packages), or paired t-tests (GraphPad Prism) were used for data analysis. Bonferroni correction was used to perform multiple comparisons correction throughout. *p* < 0.05 was considered statistically significant. Glucose uptake data was log-transformed prior statistical analysis. No power calculations were performed, and the sample sizes were not predetermined. Data collection and analysis were not blinded.

## 3. Results

**Figure 1.**
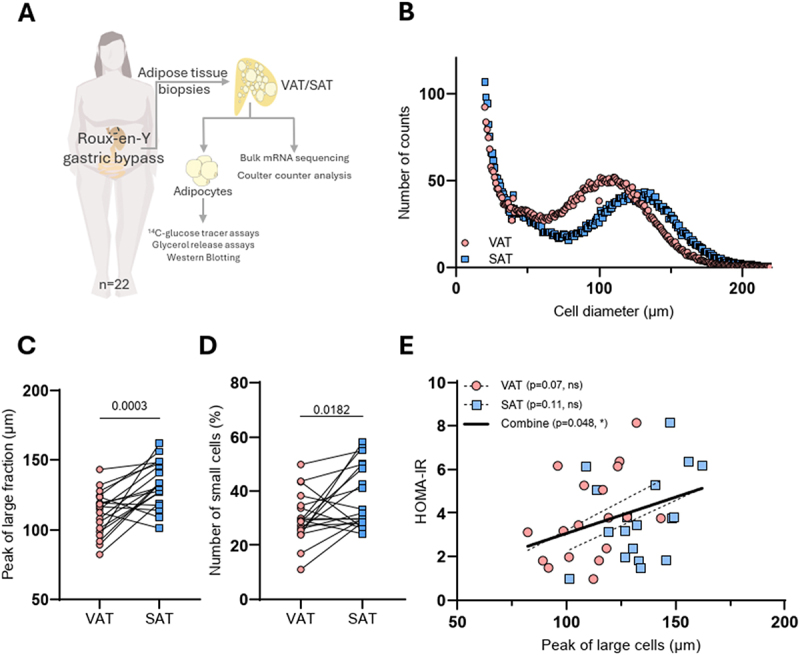
Depot differences in adipocyte size: A) Study overview: 22 patients with obesity (*n* = 4 men and *n* = 18 women) were undergoing elective Roux-en-Y gastric bypass surgery. Fat biopsies were obtained from omental-visceral white adipose tissue (VAT), and abdominal-subcutaneous white adipose tissue (SAT), used for downstream analyses in tissue (mRNA-sequencing, coulter counter) and isolated, mature adipocytes (glucose uptake, glycerol release and western blotting). (blood sample analyses and demographic information are presented in Table 1). B) Cell size distribution curves in visceral (VAT in red) and subcutaneous (SAT in blue) adipose tissue. *n* = 19. C) Peak of large cell fraction in µm in VAT and SAT samples. Statistical analysis of mean differences using a paired t-test. *n* = 19. D) Number of small cells in percentage in VAT and SAT. Statistical analysis of mean differences using a paired t-test. *n* = 19. E) Linear regression analysis showing the relationship between the peak diameter of large adipocytes (µm) and HOMA-IR (Homoeostatic model assessment of insulin resistance). *n* = 18 for VAT, *n* = 18 for SAT.

Paired SAT and VAT samples were collected from 22 patients with obesity (BMI >35, one patient BMI = 32) undergoing elective Roux-en-Y gastric bypass, intact adipose tissue samples were processed for cell size determination and bulk mRNA sequencing, and the remaining tissue underwent collagenase dissociation followed by isolation of the mature adipocyte fraction (Figure 1(A)).

Herein, we found that subcutaneous adipocytes were larger than visceral adipocytes (Figure 1(B,C)), which is well in line with previous findings [8]. A higher percentage of small adipocytes were detected in SAT compared to VAT (Figure 1(B,D)). Additionally, we found that adipocyte size, presented as peak of large cells, positively correlated with HOMA-IR of the tissue donor, meaning that patients with larger adipocytes displayed lower insulin sensitivity (Figure 1(E)). This was true irrespective of fat depot.

**Figure 2.**
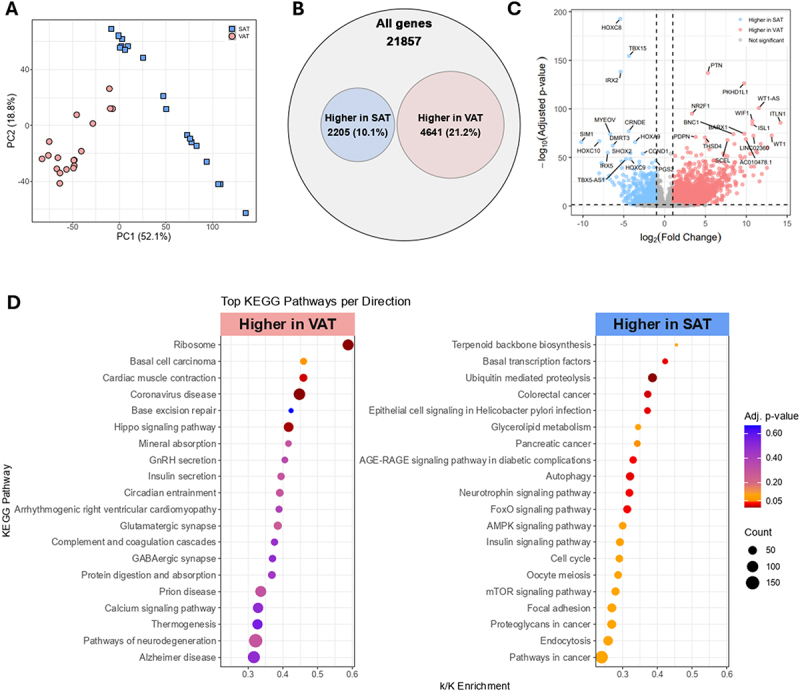
Depot differences in adipose tissue gene expression: A) PCA plot of the bulk mRNA sequencing data on paired VAT and SAT intact adipose tissue samples for the top 5000 genes. *n* = 18. B) Venn diagram showing the distribution of differentially expressed genes (DEGs) between adipose tissue depots. C) Volcano plot of differentially expressed genes comparing VAT and SAT. D) Top 20 upregulated KEGG pathways in VAT and SAT.

To further probe depot differences at the molecular level, intact SAT and VAT tissue samples from a subset (*n* = 18) of patients were analysed using bulk mRNA sequencing (RNA-Seq). Subsequent data analyses of paired VAT and SAT tissue samples showed distinct gene expression patterns in the two depots. Principal Component analysis showed that VAT-SAT differences were the major driver of PC1, explaining 52.1% of the variance, while PC2 (18.8%) captured additional within‑group variation without clearly separating the depots. Among 21,857 genes (non‑zero read counts) across all adipose tissue samples, comparison of the two depots showed that 2205 (10.1%) Differentially Expressed Genes (DEGs) were significantly higher expressed in SAT, whereas 4641 (21.2%) DEGs were significantly higher expressed in VAT (Figure 2(B)). Among these were several genes known to significantly differ between depots, such as SHOX2 for SAT, NR2F1 and WT1 for VAT (Figure 2(C)) [28]. KEGG-pathway enrichment analysis of these DEGs showed elevated pro-oncogenic and cell cycle pathways especially in SAT, but also increased pathways related to focal adhesion, endocytosis, insulin signalling, and AMPK signalling (Figure 2(D)). In contrast, VAT showed clear enrichment in ribosome-related KEGG pathways and expression patterns related to neurological conditions and the Hippo signalling pathway, the latter involved in mechano-sensing (Figure 2(D)).

**Figure 3.**
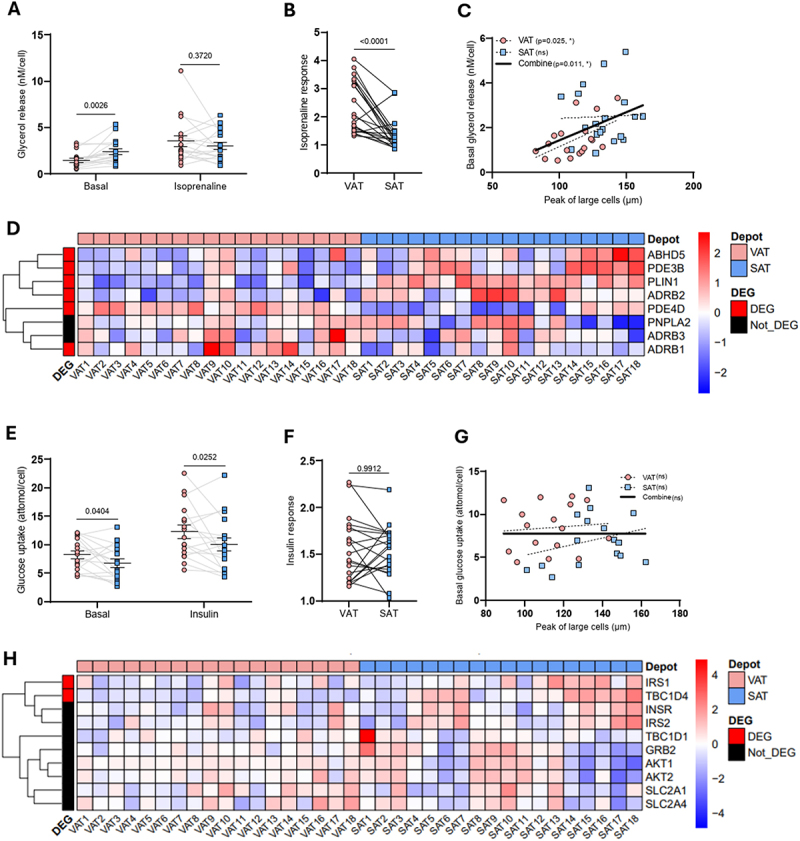
Depot differences in adipocyte glucose uptake and lipolysis: A) Basal and isoprenaline-stimulated glycerol release per cell in collagenase-isolated adipocytes of VAT and SAT samples (VAT in red, SAT in blue). Statistical analysis was performed using two-way repeated measures ANOVA and post-hoc multiple comparisons with the Bonferroni correction. *n* = 18. B) Isoprenaline response was calculated as the fold response of isoprenaline over basal glycerol release in collagenase-isolated adipocytes. Statistical analysis of geometric mean differences was performed using a ratio paired t-test. *n* = 21. C) Linear regression analysis showing the relationship between the peak diameter of large adipocytes (µm) and basal glycerol release per cell. *n* = 18. D) Heatmap of lipolysis-related gene targets highlighting depot differences in paired analysis of SAT and VAT samples. *n* = 18. E) Basal and insulin-stimulated glucose uptake per cell in isolated adipocytes of VAT and SAT samples. Statistical analysis was performed using two-way repeated measures ANOVA and post-hoc multiple comparisons with the Bonferroni correction. *n* = 17. Glucose uptake data was log-transformed prior analysis. F) Insulin response calculated as the fold response of insulin over basal glucose uptake. Statistical analysis of geometric mean differences was performed using a ratio paired t-test. *n* = 20. G) Linear regression analysis showing the relationship between the peak diameter of large adipocytes (µm) and basal glucose uptake per cell. *n* = 16. H) Heatmap of insulin signalling-related gene targets highlighting depot differences in paired analysis of SAT and VAT adipose tissue samples. *n* = 18. Data in A and E displayed as mean ± SEM.

Next, we functionally characterized and compared collagenase-isolated adipocytes from the two fat depots with a focus on identifying depot-specific differences. By measuring basal and isoprenaline-induced lipolysis, we found that isoprenaline responsiveness (fold increase) was higher for VAT adipocytes than SAT adipocytes (Figure 3(A,B)). Basal lipolysis was higher in SAT adipocytes compared to VAT adipocytes (Figure 3(A)). The rate of basal lipolysis correlated positively with adipocyte size in VAT but not SAT adipocytes (Figure 3(C)). To further probe the underlying molecular cause behind this difference, we analysed the collected RNA-Seq data set for targets known to regulate lipolytic events. Indeed, we found that the gene expression levels of *ABHD5*, *PDE3B*, *PLIN1* and *ADRB2* were higher in SAT compared with VAT (Figure 3(D)). Additionally, we assessed insulin responsiveness in the same cell preparation using glucose tracer assays. Both basal and insulin-stimulated glucose uptake were higher in VAT than SAT adipocytes comparing each individual (Figure 3(E)), but no depot-specific differences were observed when plotted as insulin responsiveness (fold increase) (Figure 3(F)). Peak of large cells did not hold any predictive power for basal glucose uptake (Figure 3(G)), irrespective of depot origin. Subsequent RNA-Seq data analysis of genes known to regulate glucose transport showed that only *IRS1* and *TBC1D4* levels were significantly higher in SAT compared to VAT (Figure 3(H)).

**Figure 4.**
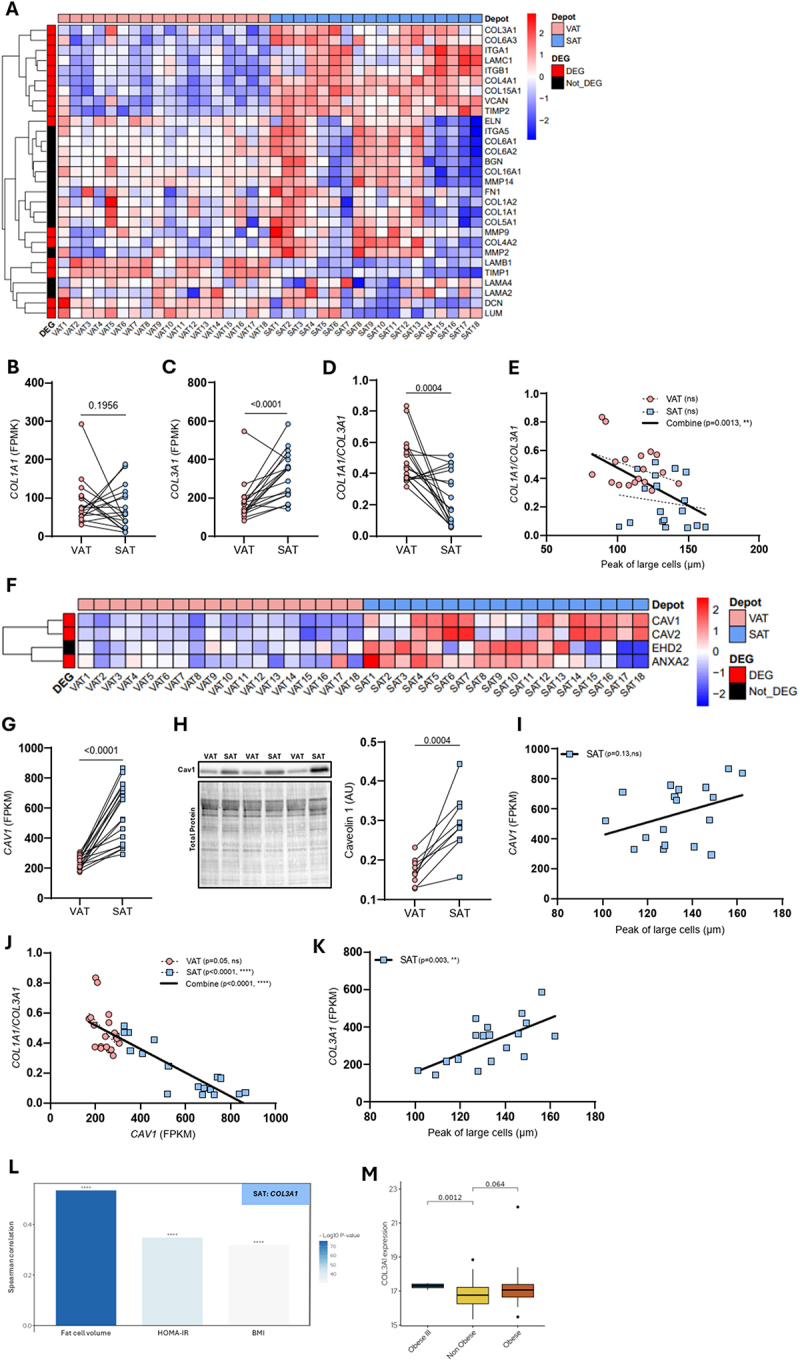
Depot differences in extracellular matrix composition, caveolin content and the link to systemic insulin sensitivity: A) Heatmap of extracellular matrix-related gene targets highlighting depot differences in paired analysis of SAT and VAT adipose tissue samples. *n* = 18. B) COL1A1 gene expression (bulk mRNA sequencing) in VAT and SAT adipose tissue samples. *n* = 18. C) COL3A1 gene expression (bulk mRNA sequencing) in VAT and SAT adipose tissue samples. *n* = 18. D) Ratio of COL1A1/COL3A1 gene expression in VAT and SAT adipose tissue samples. Statistical analysis of geometric mean differences was performed using a ratio paired t-test. *n* = 18. E) linear regression analysis showing the relationship between the peak diameter of large adipocytes (µm) and the COL1A1/COL3A1 ratio. *n* = 18. F) Heatmap of caveolae-related gene targets highlighting depot differences in paired analysis of SAT and VAT adipose tissue samples. *n* = 18. G) CAV1 gene expression (bulk mRNA sequencing) in VAT and SAT adipose tissue samples. *n* = 18. H) Quantification and representative western blot for the analysis of Caveolin 1 protein levels in collagenase-isolated adipocyte lysates. Statistical analysis of geometric mean differences was performed using a ratio paired t-test. *n* = 9. I) Linear regression analysis showing the relationship between the peak diameter of large adipocytes (µm) and CAV1 in SAT adipose tissue. *n* = 18. J) Linear regression analysis showing the relationship between CAV1 and COL1A1/COL3A1 in adipose tissue. *n* = 18. K) Linear regression analysis showing the relationship between the peak diameter of large adipocytes (µm) and COL3A1 in SAT adipose tissue. *n* = 18. L) Spearman correlation of COL3A1 expression in subcutaneous adipose tissue and fat cell volume, HOMA-IR, and BMI. Data derived from [29]. M) COL3A1 expression in adipose tissue from patients without obesity (BMI < 30), patients with obesity (BMI >30), and patients with obesity III (BMI >40). Data derived from [29].

Next, considering the literature showing fat depot-specific differences in extracellular matrix composition [17–19], we analysed the expression levels of known ECM targets in our collected RNA-Seq data set. We found significantly higher expression levels of several ECM marker genes in SAT compared to VAT (Figure 4(A)). Amongst those, we were specifically interested in collagens that previously have been shown to influence tissue expandability, stiffness and insulin resistance [18]. We found higher expression levels of *COL3A1*, *COL6A3*, *COL4A1*, *COL15A1*, and *COL4A2* in SAT, while *COL1A1* expressions were similar in both depots (Figure 4(A-C)). In various tissues, *COL1A1/COL3A1* ratio has been used as a proxy for ECM stiffness vs flexibility, where a higher ratio corresponds to a stiffer tissue phenotype [30–34]. Here, we found that the *COL1A1/COL3A1* ratio was significantly lower in SAT than VAT (Figure 4(B-D)). Interestingly, the peak of large adipocytes (combined analysis) negatively correlated with *COL1A1/COL3A1* ratio, suggesting larger adipocyte sizes are associated with reduced tissue stiffness across depots (Figure 4(E)).

Further RNA-Seq data analysis showed higher mRNA expression levels of Caveolin 1 (*CAV1*), the core protein of specific plasma-membrane domains, caveolae, in SAT compared to VAT (Figure 4(F,G)). This finding was confirmed at the protein level using adipocyte lysates (Figure 4(H)). The depot-specific differences of both *COL3A1* and *CAV1* expression were also observed in a larger cohort of comparable adipose tissue samples (Figure S1A, Data: [29,35–49]). Moreover, gene expression of the caveolae-associated proteins *CAV2* and *ANXA2* were also higher in SAT than in VAT (Figure 4(F)). Caveolae can serve as a contextual indicator of membrane flexibility, supported by recent studies showing that caveolae-deficient adipose tissue is stiffer, less deformable, and prone to rupture under compression [50] and another study demonstrating that caveolin 1 expression serves as an important predictor of cellular expandability in response to overfeeding [51]. Our findings could possibly reflect a higher caveolae density and an increased adipose expansion capacity in SAT adipocytes vs VAT adipocytes. In support, we found a trend for positive correlation between *CAV1* expression and SAT adipocyte size (Figure 4(I)). Intriguingly, *CAV1* expression showed a negative correlation with *COL1A1/COL3A1* (Figure 4(J)).

To test if our findings could be informative for understanding the contribution of adipose tissue stiffness to systemic metabolism, we investigated the association of *COL3A1* expression with adipocyte size, HOMA-IR, and BMI. Strikingly, we found that the peak of large cells showed a positive correlation with *COL3A1* levels in SAT (Figure 4(K)), while no correlation was observed in VAT (Figure S1B). Furthermore, we observed a weak positive correlation (not significant) between *COL3A1* and the BMI and HOMA-IR of the biopsy donors for SAT but not in VAT (Figure S1C-F). This was further supported by data from a larger, publicly available cohort of adipose tissue samples, where SAT *COL3A1* expression positively correlated with both fat cell volume and the HOMA-IR and BMI of the donors (Figure 4(L) and Figure S1G, Data [29]:). In agreement, in that data set, *COL3A1* was more highly expressed in adipose tissue of people with obesity (BMI >30), and people with obesity III (BMI >40) compared with people without obesity (BMI < 30) (Figure 4(M), Data [29]:).

Taken together, the increased *COL3A1* expression in patients with obesity and the positive correlation between *COL3A1*, adipocyte size, and HOMA-IR in specifically SAT, suggest *COL3A1* as a mediator of adipose tissue flexibility and expandability. It further underlines the potential contribution of adipose tissue dynamics in obesity to systemic insulin resistance.

## 4. Discussion

This study provides a characterization of key functional and molecular differences between abdominal SAT and omental VAT in individuals with obesity. Our findings highlight depot-specific discrepancies in adipocyte size, lipolytic activity, glucose uptake, and factors potentially limiting adipocyte expansion. They expand previous knowledge of key depot differences in humans by using paired SAT and VAT samples from the same individuals throughout the study, enabling comparison not only across depots but also across different types of assays, such as functional measurements and RNA-Seq data. In addition, few previous studies have provided multiple functional analyses of adipocytes from both depots of the same individuals. Together, this comprehensive mapping of key features of human adipose tissue function in obesity can be used as a basis for further mechanistic studies.

Consistent with previous reports [8,52,53] we observed that SAT adipocytes were significantly larger than VAT adipocytes in subjects with obesity. Herein, we report a higher percentage of small cells in SAT than VAT, suggesting also a higher level of spare capacity for lipid storage in this depot. In contrast, Liu. *et al*. found no depot differences in the abundance of small adipocytes [54]. These discrepancies likely originate from differences in patient characteristics, e.g. degree of obesity, sex, and age, or ethnicity or technical differences in the method of cell size measurement. In accordance with previous studies, we show that adipocyte size is associated with systemic insulin resistance as measured by the surrogate marker HOMA-IR [8,55].

The functional assays showed that in contrast to previous reports [11], basal lipolysis was higher in SAT than in VAT adipocytes. However, catecholamine responsiveness was higher in VAT than in SAT adipocytes, which is consistent with previous findings [11,56]. The significant association between fat cell-size and basal lipolysis in VAT adipocytes agrees with a detrimental role of hypertrophic VAT expansion for systemic health during obesity progression. Possibly, the higher spontaneous lipolysis in SAT adipocytes reflects an impairment of insulin’s antilipolytic effect. In agreement with earlier reports [57,58], we found that basal- and insulin-stimulated glucose uptake were higher in VAT than in SAT adipocytes, which agrees with earlier findings rendering VAT adipocytes as more metabolically active compared to SAT in the obese state [59]. Interestingly, glucose uptake was not correlated to adipocyte size, suggesting that the observed depot differences are intrinsic to each depot, and not driven by differences in adipocyte lipid storage.

Using bulk mRNA sequencing, we found distinct gene expression patterns comparing VAT and SAT. Among the most significant DEGs, we observed several established transcriptional markers for SAT and VAT [28], providing external validity to our results. Furthermore, we found an enrichment in pro-oncogenic and cell cycle related KEGG pathways especially in SAT, which is in line with previous findings showing that SAT more readily undergoes endoreplication than VAT, explored in detail by Li and Hagberg *et al.* [60]. We also found that KEGG pathways related to AMPK, mTOR and insulin signalling were more highly expressed in SAT than in VAT. This is also in line with previous studies showing higher AMPK activity in SAT compared to VAT of patients with obesity [61], and in patients with obesity and preserved insulin sensitivity (maintained low HOMA-IR [61]).

While the association between increased basal lipolysis and higher expression of lipolysis-related genes in SAT was expected, the upregulated gene expression of *IRS1* and *TBC1D4* were somewhat contradictory to the observed reduction in glucose transport in this depot. The relatively higher expression levels of *COL3A1, COL6A3, COL4A1*, and *COL15A1* in SAT are in line with previous studies [17,18,62]. COL1A1 forms thick fibres that increase tissue stiffness, whereas COL3A1 forms thin, reticular fibres that enhance elasticity. Previous studies across various tissues have shown that the balance between these collagens may serve as an indicator of tissue expandability [30,33]. Changes in the collagen I-to-collagen III ratio have been linked to myocardial stiffness [32] and increased postoperative scarring [31]. In patients with cerebral palsy the collagen I/III ratio was a key predictor of muscle tissue stiffness [34]. Mechanistic studies demonstrated the possible relationship between increased collagen I-to-collagen III ratio and increased fibril stiffness [63,64]. Given that adipose tissue fibrosis and impaired ECM remodelling are linked to adipocyte dysfunction [1], our findings suggest that despite SAT expressing higher levels of ECM transcripts overall, VAT may be more prone to fibrotic changes and tissue stiffness, further exacerbating metabolic dysregulation. The fact that we found higher Caveolin 1 expression in SAT, both at the gene and protein level, together with a robust correlation between *COL1A1/COL3A1* and Caveolin 1, as well as *COL3A1* and adipocyte size argue for lower tissue stiffness and higher adipocyte and tissue flexibility in SAT relative to VAT. These characteristics could allow SAT adipocytes to grow larger in size and store more lipids than VAT adipocytes, which could contribute to reduced ectopic lipid deposition. In line with that a recent study demonstrated that caveolae contribute to adipocyte expandability by supporting membrane flexibility during lipid droplet growth, with caveolin 1 deficiency being associated with reduced lipid-loading capacity and increased cellular and tissue stiffness [50]. Accordingly, a study by Briand *etal.* highlights that caveolin 1 expression as an important determinant of caveolae density, adipocyte lipid droplet growth and as a predictor of cellular expandability in response to overfeeding [51]. These results underline a need for further studies exploring the correlation between *COL3A1* expression with parameters of tissue stiffness, cellular function and metabolic health, focusing especially on SAT storage capacity. This is especially important considering the major posttranslational modifications collagens undergo, which influence tissue properties [65]. Additionally, we found *COL3A1* to positively correlate with fat cell size, BMI, and HOMA-IR, which highlights a possible role of tissue stiffness and COL3A1 for adipocyte expansion but also for obesity-associated insulin resistance. In a weight-discordant twin study on adipose tissue, Kaartinen *et al.* found that SAT *COL3A1* expression showed a positive correlation to liver fat and HOMA-IR [66]. In cultured adipocytes, Al-Hasan *et al.* demonstrated that COL3A1 plays an important role in adipogenesis [67]. Taken together, this underscores the potential role of COL3A1 in promoting insulin resistance in obesity.

In summary, by combining functional analyses with transcriptomic characterization we provide evidence supporting distinct metabolic properties of subcutaneous and visceral adipocytes and tissue in patients with obesity. Our data demonstrates that the extracellular environment in SAT and caveolae-related characteristics support beneficial adipose tissue expansion, whereas VAT adipocytes display higher metabolic activity and features consistent with reduced expandability. These findings significantly expand previous knowledge of depot-specific differences in humans. The combined approach examining both cellular function and tissue characteristics, together with depot-comparison in the same individual exerts a strength and important contribution to the research field that serves as a foundation for further mechanistic studies.

### 4.1. Limitations

The patients included in this study were individuals with obesity, and due to the limited sample size, we could not account for sex- and age-specific effects on adipose tissue. Yet, confounding factors should only play a limited role in the paired depot comparison conducted herein. Notably, we analysed glucose uptake and glycerol release in isolated adipocytes (*ex vivo*) and not *in vivo*. However, previous findings indicate that *ex vivo*-measured glucose transport serves as a tool for cellular insulin sensitivity *in vivo* [68], and that *ex vivo* glycerol release is a reliable method for estimating *in vivo* adipose tissue function [69]. Adenosine was included in the glucose uptake and lipolysis assay and could theoretically affect the results. However, all samples were treated equally; therefore, any potential effect would be expected to affect SAT and VAT adipocytes similarly and should not interfere with the observed depot-specific differences in adipocyte lipolysis. Due to technical limitations, cell lysates of isolated adipocytes for western blot analysis were only obtained for nine patients. Notably, bulk mRNA sequencing of adipose tissue captures gene expression not only from adipocytes but also from other adipose-resident cell types. Furthermore, intact adipose tissue was used for cell size determination, and therefore artefacts (broken cells, lipid droplets) and other cell types could influence the result. This was handled by adjusting the lower cut-off to ensure only intact mature adipocytes were measured. Notably, collagen mRNA expression is merely a proxy of tissue stiffness, and further validation of tissue properties is warranted in future studies.

## Supplementary Material

Supplemental Material

## Data Availability

Data that support the findings are available in Figshare, at 10.6084/m9.figshare.32090950.

## References

[1] Hagberg CE, Spalding KL. White adipocyte dysfunction and obesity-associated pathologies in humans. Nat Rev Mol Cell Biol 25, 270–289. 2023. doi: 10.1038/s41580-023-00680-1 38086922

[2] Börgeson E, Boucher J, Hagberg CE. Of mice and men: pinpointing species differences in adipose tissue biology. Front Cell Dev Biol. 2022 Sep 15;10:1003118. 2022. doi: 10.3389/fcell.2022.1003118 PMC9521710 36187476

[3] Chusyd DE, Wang D, Huffman DM. Relationships between rodent white adipose fat pads and human white adipose fat depots. Front Nutr. 2016 Apr 19;3:10. 2016. doi: 10.3389/fnut.2016.00010 PMC4835715 27148535

[4] Kwon H, Kim D, Kim JS. Body fat distribution and the risk of incident metabolic syndrome: a longitudinal cohort study. Sci Rep. 2017;7(1). doi: 10.1038/s41598-017-09723-y PMC5591218 28887474

[5] Neeland IJ, Ayers CR, Rohatgi AK, et al. Associations of visceral and abdominal subcutaneous adipose tissue with markers of cardiac and metabolic risk in obese adults. Obesity. 2013;21(9). doi: 10.1002/oby.20135 PMC3751977 23687099

[6] Tchoukalova YD, Votruba SB, Tchkonia T, et al. Regional differences in cellular mechanisms of adipose tissue gain with overfeeding. Proc Natl Acad Sci U S A. 2010;107(42):18226–15. doi: 10.1073/pnas.1005259107 20921416 PMC2964201

[7] Laurencikiene J, Skurk T, Kulyté A, et al. Regulation of lipolysis in small and large fat cells of the same subject. J Clin Endocrinol Metab. 2011;96(12):E2045–E2049. doi: 10.1210/jc.2011-1702 21994963

[8] Ye RZ, Richard G, Gévry N, et al. Fat cell size: measurement methods, pathophysiological origins, and relationships with metabolic dysregulations. Endocr Rev. 2022;43(1):35–60. doi: 10.1210/endrev/bnab018 34100954 PMC8755996

[9] Rydén M, Arner P. Subcutaneous adipocyte lipolysis contributes to circulating lipid levels. Arterioscler Thromb Vasc Biol. 2017;37(9):1782–1787. doi: 10.1161/ATVBAHA.117.309759 28663255 PMC5567402

[10] Rebuffé-Scrive M, Anderson B, Olbe L, et al. Metabolism of adipose tissue in intraabdominal depots in severely obese men and women. Metabolism. 1990;39(10):1021–1025. doi: 10.1016/0026-0495(90)90160-E 2215250

[11] Arner P. Differences in lipolysis between human subcutaneous and omental adipose tissues. Ann Med. 1995 Aug;27(4):435-8. 1995. doi: 10.3109/07853899709002451 8519504

[12] Koutsari C, Jensen MD. Thematic review series: patient-oriented Research. Free fatty acid metabolism in human obesity. J Lipid Res. 2006;47(8):1643–1650. doi: 10.1194/jlr.r600011-jlr200 16685078

[13] Jensen MD. Role of body fat distribution and the metabolic complications of obesity. J Clin Endocrinol Metab. 2008 Nov;93(11 Suppl 1):S57-63. 2008. doi: 10.1210/jc.2008-1585 PMC2585758 18987271

[14] Divoux A, Tordjman J, Lacasa D, et al. Fibrosis in human adipose tissue: composition, distribution, and link with lipid metabolism and fat mass loss. Diabetes. 2010;59(11):2817–2825. doi: 10.2337/db10-0585 20713683 PMC2963540

[15] Marcelin G, Gautier EL, Clement K. Adipose tissue fibrosis in obesity: etiology and challenges. Annu Rev Physiol. 2022 Feb 10;84:135–155. doi: 10.1146/annurev-physiol-060721-092930 34752708

[16] Verboven K, Wouters K, Gaens K, et al. Abdominal subcutaneous and visceral adipocyte size, lipolysis and inflammation relate to insulin resistance in male obese humans. Sci Rep. 2018;8(1). doi: 10.1038/s41598-018-22962-x PMC5856747 29549282

[17] Reggio S, Rouault C, Poitou C, et al. Increased basement membrane components in adipose tissue during obesity: links with TGF- And metabolic phenotypes. J Clin Endocrinol Metab. 2016;101(6). doi: 10.1210/jc.2015-4304 27049236

[18] Soták M, Rajan MR, Clark M, et al. Healthy subcutaneous and omental adipose tissue is associated with high expression of extracellular matrix components. Int J Mol Sci. 2022;23(1):520. doi: 10.3390/ijms23010520 35008946 PMC8745535

[19] Ruiz-Ojeda FJ, Méndez-Gutiérrez A, Aguilera CM. *et al*. Extracellular matrix remodeling of adipose tissue in obesity and metabolic diseases. Int J Mol Sci. 2019 Oct 2;20(19):4888. doi: 10.3390/ijms20194888 PMC6801592 31581657

[20] Mathur N, Severinsen MCK, Jensen ME, et al. Human visceral and subcutaneous adipose stem and progenitor cells retain depot-specific adipogenic properties during obesity. Front Cell Dev Biol. 2022;10. doi: 10.3389/fcell.2022.983899 PMC9629396 36340033

[21] Manolopoulos KN, Karpe F, Frayn KN. Gluteofemoral body fat as adeterminant of metabolic health. Int J Obes, 2010;34949–959. doi: 10.1038/ijo.2009.286 20065965

[22] Matthews DR, Hosker JP, Rudenski AS, et al. Homeostasis model assessment: insulin resistance and β-cell function from fasting plasma glucose and insulin concentrations in man. Diabetologia. 1985;28(7):412–419. doi: 10.1007/BF00280883 3899825

[23] Rodbell M. Metabolism of isolated fat cells. I. Effects of hormones on glucose metabolism and lipolysis. J Biol Chem. 1964;239(2):375–380. doi: 10.1016/S0021-9258(18)51687-2 14169133

[24] McLaughlin T, Lamendola C, Coghlan N, et al. Subcutaneous adipose cell size and distribution: relationship to insulin resistance and body fat. Obesity. 2014;22(3):673–680. doi: 10.1002/oby.20209 23666871 PMC4344365

[25] Jo J, Gavrilova O, Pack S, et al. Hypertrophy and/or hyperplasia: dynamics of adipose tissue growth. PLoS Comput Biol. 2009;5(3):e1000324. doi: 10.1371/journal.pcbi.1000324 19325873 PMC2653640

[26] Gliemann J, Rees WD, Foley JA. The fate of labelled glucose molecules in the rat adipocyte. Dependence on glucose concentration. BBA Mol Cell Res. 1984;804(1):68–76. doi: 10.1016/0167-4889(84)90100-9 6372867

[27] R Core Team. R: a language and environment for statistical computing. 2019.

[28] Harms MJ, Li Q, Lee S, et al. Mature human white adipocytes cultured under membranes maintain identity, function, and can transdifferentiate into brown-like adipocytes. Cell Rep. 2019;27(1):213–225.e5. doi: 10.1016/j.celrep.2019.03.026 30943403

[29] Zhong J, Zareifi D, Weinbrenner S, et al. Adiposetissue.Org: a knowledge portal integrating clinical and experimental data from human adipose tissue. Cell Metab. 2025;37(3):566–569. doi: 10.1016/j.cmet.2025.01.012 39983713

[30] Kanniyappan H, Chathurika Rathnayake RA, Osamor J *et al*. The role of collagen and collagen I/III ratio in pathological conditions: insights into molecular mechanisms and therapeutic approaches. Front Bioeng Biotechnol. 2025 Oct 9;13:1679625. doi: 10.3389/fbioe.2025.1679625 PMC12548377 41141281

[31] Kim HY, Im H-Y, Chang H-K, et al. Correlation between collagen type I/III ratio and scar formation in patients undergoing immediate reconstruction with the round block technique after breast-conserving surgery. Biomedicines. 2023;11(4):1089. doi: 10.3390/biomedicines11041089 37189707 PMC10135880

[32] Marijianowski MMH, Teeling P, Mann J, et al. Dilated cardiomyopathy is associated with an increase in the type I/type III collagen ratio: a quantitative assessment. J Am Coll Cardiol. 1995;25(6):1263–1272. doi: 10.1016/0735-1097(94)00557-7 7722119

[33] Singh D, Rai V, Agrawal DK. Regulation of collagen I and collagen III in tissue injury and regeneration. Cardiol Cardiovasc Med. 2023;7(1). doi: 10.26502/fccm.92920302 PMC9912297 36776717

[34] Smith LR, Pichika R, Meza RC, et al. Contribution of extracellular matrix components to the stiffness of skeletal muscle contractures in patients with cerebral palsy. Connect Tissue Res. 2021;62(3):287–298. doi: 10.1080/03008207.2019.1694011 31779492 PMC7253322

[35] Du Plessis J, van Pelt J, Korf H, et al. Association of adipose tissue inflammation with histologic severity of nonalcoholic fatty liver disease. Gastroenterology. 2015;149(3):635–648.e14. doi: 10.1053/j.gastro.2015.05.044 26028579

[36] Johansson LE, Danielsson AP, Parikh H, et al. Differential gene expression in adipose tissue from obese human subjects during weight loss and weight maintenance. Am J Clin Nutr. 2012;96(1):196–207. doi: 10.3945/ajcn.111.020578 22648723

[37] Salcedo-Tacuma D, Bonilla L, Montes MCG, et al. Transcriptome dataset of omental and subcutaneous adipose tissues from gestational diabetes patients. Sci Data. 2022;9(1). doi: 10.1038/s41597-022-01457-5 PMC9205947 35715414

[38] Vink RG, Roumans NJ, Fazelzadeh P, et al. Adipose tissue gene expression is differentially regulated with different rates of weight loss in overweight and obese humans. Int J Obes. 2017;41(2):309–316. doi: 10.1038/ijo.2016.201 27840413

[39] Winnier DA, Fourcaudot M, Norton L, et al. Transcriptomic identification of adh1b as a novel candidate gene for obesity and insulin resistance in human adipose tissue in Mexican americans from the veterans administration genetic epidemiology study (vages). PLOS ONE. 2015;10(4):e0119941. doi: 10.1371/0119941 25830378 PMC4382323

[40] Armenise C, Lefebvre G, Carayol J, et al. Transcriptome profiling from adipose tissue during a low-calorie diet reveals predictors of weight and glycemic outcomes in obese, nondiabetic subjects. Am J Clin Nutr. 2017;106(3):736–746. doi: 10.3945/ajcn.117.156216 28793995

[41] Imbert A, Vialaneix N, Marquis J, et al. Network analyses reveal negative link between changes in adipose tissue GDF15 and BMI during dietary-induced weight loss. J Clin Endocrinol Metab. 2022;107(1):e130–e142. doi: 10.1210/clinem/dgab621 34415992

[42] Arner P, Sahlqvist A-S, Sinha I, et al. The epigenetic signature of systemic insulin resistance in obese women. Diabetologia. 2016;59(11):2393–2405. doi: 10.1007/s00125-016-4074-5 27535281 PMC5506095

[43] Civelek M, Wu Y, Pan C, et al. Genetic regulation of adipose gene expression and Cardio-metabolic traits. Am J Hum Genet. 2017;100(3):428–443. doi: 10.1016/j.ajhg.2017.01.027 28257690 PMC5339333

[44] Raulerson CK, Ko A, Kidd JC, et al. Adipose tissue gene expression associations reveal hundreds of Candidate genes for cardiometabolic traits. Am J Hum Genet. 2019;105(4):773–787. doi: 10.1016/j.ajhg.2019.09.001 31564431 PMC6817527

[45] Stančáková A, Civelek M, Saleem NK, et al. Hyperglycemia and a common variant of GCKR are associated with the levels of eight amino acids in 9,369 Finnish men. Diabetes. 2012;61(7):1895–1902. doi: 10.2337/db11-1378 22553379 PMC3379649

[46] Arner E, Mejhert N, Kulyté A, et al. Adipose tissue MicroRNAs as regulators of CCL2 production in human obesity. Diabetes. 2012;61(8):1986–1993. doi: 10.2337/db11-1508 22688341 PMC3402332

[47] Arner P, Andersson DP, Bäckdahl J, et al. Weight gain and impaired glucose metabolism in women are predicted by inefficient subcutaneous fat cell lipolysis. Cell Metab. 2018;28(1):45–54.e3. doi: 10.1016/j.cmet.2018.05.004 29861390

[48] Petrus P, Mejhert N, Corrales P, et al. Transforming growth factor-β3 regulates adipocyte number in subcutaneous white adipose tissue. Cell Rep. 2018;25(3):551–560.e5. doi: 10.1016/j.celrep.2018.09.069 30332637

[49] Kerr AG, Andersson DP, Rydén M, et al. Long-term changes in adipose tissue gene expression following bariatric surgery. J Intern Med. 2020;288(2):219–233. doi: 10.1111/joim.13066 32406570

[50] Aboy-Pardal MCM, Guadamillas MC, Guerrero CR, et al. Plasma membrane remodeling determines adipocyte expansion and mechanical adaptability. Nat Commun. 2024;15(1):10102. doi: 10.1038/s41467-024-54224-y 39609408 PMC11605069

[51] Briand N, Prado C, Mabilleau G, et al. Caveolin-1 expression and cavin stability regulate caveolae dynamics in adipocyte lipid store fluctuation. Diabetes. 2014;63(12):4032–4044. doi: 10.2337/db13-1961 24969108 PMC4238006

[52] Van Harmelen V, Reynisdottir S, Eriksson P, et al. Leptin secretion from subcutaneous and visceral adipose tissue in women. Diabetes. 1998;47(6):913–917. doi: 10.2337/diabetes.47.6.913 9604868

[53] Despres JP, Fong BS, Julien P, et al. Regional variation in HDL metabolism in human fat cells: effect of cell size. Am J Physiol Endocrinol Metab. 1987;252(15/5):E654–E659. doi: 10.1152/ajpendo.1987.252.5.e654 3578514

[54] Liu A, McLaughlin T, Liu T, et al. Differential intra-abdominal adipose tissue profiling in obese, insulin-resistant women. Obes Surg. 2009;19(11):1564–1573. doi: 10.1007/s11695-009-9949-9 19711137 PMC3181138

[55] Stenkula KG, Erlanson-Albertsson C. Adipose cell size: importance in health and disease. Am J Physiol Regul Integr Comp Physiol. 2018 Aug 1;315(2):R284-R295. doi: 10.1152/ajpregu.00257.2017 29641234

[56] Hellmér J, Marcus C, Sonnenfeld T, et al. Mechanisms for differences in lipolysis between human subcutaneous and omental fat cells. J Clin Endocrinol Metab. 1992;75(1):15–20. doi: 10.1210/jcem.75.1.1320047 1320047

[57] Christen T, Sheikine Y, Rocha VZ, et al. Increased glucose uptake in visceral versus subcutaneous adipose tissue revealed by PET imaging. JACC Cardiovasc Imag. 2010;3(8):843–851. doi: 10.1016/j.jcmg.2010.06.004 PMC4042675 20705265

[58] Virtanen KA, Lönnroth P, Parkkola R, et al. Glucose uptake and perfusion in subcutaneous and visceral adipose tissue during insulin stimulation in nonobese and obese humans. J Clin Endocrinol Metab. 2002;87(8):3902–3910. doi: 10.1210/jcem.87.8.8761 12161530

[59] Ibrahim MM. Subcutaneous and visceral adipose tissue: structural and functional differences. Obes Rev. 2010 Jan;11(1):11-8. doi: 10.1111/j.1467-789X.2009.00623.x 19656312

[60] Li Q, Hagberg CE, Silva Cascales H, et al. Obesity and hyperinsulinemia drive adipocytes to activate a cell cycle program and senesce. Nat Med. 2021;27(11):1941–1953. doi: 10.1038/s41591-021-01501-8 34608330

[61] Gauthier MS, O’Brien EL, Bigornia S, et al. Decreased AMP-activated protein kinase activity is associated with increased inflammation in visceral adipose tissue and with whole-body insulin resistance in morbidly obese humans. Biochem Biophys Res Commun. 2011;404(1):382–387. doi: 10.1016/j.bbrc.2010.11.127 21130749 PMC3061625

[62] McCulloch LJ, Rawling TJ, Sjöholm K, et al. COL6A3 is regulated by leptin in human adipose tissue and reduced in obesity. Endocrinology. 2015;156(1):134–146. doi: 10.1210/en.2014-1042 25337653

[63] Li W, Chi N, Rathnayake RAC, et al. Distinctive roles of fibrillar collagen I and collagen III in mediating fibroblast-matrix interaction: a nanoscopic study. Biochem Biophys Res Commun. 2021;560. doi: 10.1016/j.bbrc.2021.04.088 PMC8165026 33975247

[64] Asgari M, Latifi N, Heris HK, et al. In vitro fibrillogenesis of tropocollagen type III in collagen type i affects its relative fibrillar topology and mechanics. Sci Rep. 2017;7(1). doi: 10.1038/s41598-017-01476-y PMC5431193 28469139

[65] Lindquist JN, Marzluff WF, Stefanovic B. Fibrogenesis III. Posttranscriptional regulation of type Icollagen Am J Physiol Gastrointest Liver Physiol. 2000 Sep;279(3):G471-6. doi: 10.1152/ajpgi.2000.279.3.g471 10960344

[66] Kaartinen MT, Hang A, Barry A, et al. Matrisome alterations in obesity – adipose tissue transcriptome study on monozygotic weight-discordant twins. Matrix Biol. 2022;108:1–19. doi: 10.1016/j.matbio.2022.02.005 35227930

[67] Al Hasan M, Martin PE, Shu X, et al. Type III collagen is required for adipogenesis and actin stress fibre formation in 3T3-L1 preadipocytes. Biomolecules. 2021;11(2):156. doi: 10.3390/biom11020156 33504048 PMC7911635

[68] Kashiwagi A, Verso MA, Andrews J, et al. In vitro insulin resistance of human adipocytes isolated from subjects with noninsulin-dependent diabetes mellitus. J Clin Invest. 1983;72(4):1246–1254. doi: 10.1172/JCI111080 6355180 PMC370408

[69] Arner P. Human fat cell lipolysis: biochemistry, regulation and clinical role. Best Pract Res Clin Endocrinol Metab. 2005 Dec;19(4):471–82. doi: 10.1016/j.beem.2005.07.004 16311212

