# Supplementary figures and images for: Depot-specific differences in visceral and subcutaneous adipose tissue from patients with obesity
Source: Adipocyte. 2026 Jul 26;15(1):2708374. doi: 10.1080/21623945.2026.2708374 (PMC13418479; doi:10.1080/21623945.2026.2708374)

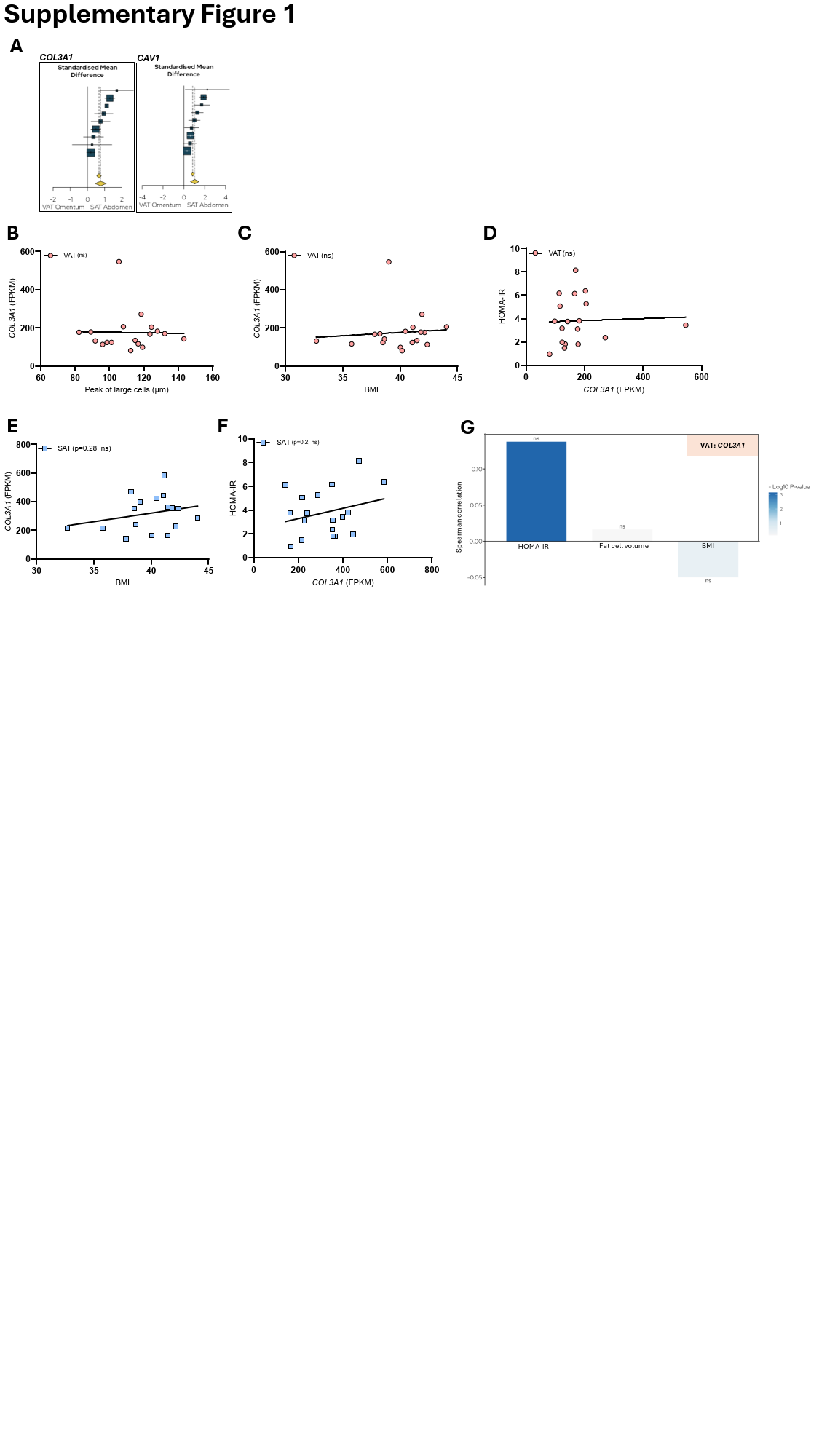

Supplement: Supplemental Material [file KADI_A_2708374_SM9622.tif]
